# Mutual Regulation of NOD2 and RIG-I in Zebrafish Provides Insights into the Coordination between Innate Antibacterial and Antiviral Signaling Pathways

**DOI:** 10.3390/ijms18061147

**Published:** 2017-05-27

**Authors:** Li Nie, Xiao-Xiao Xu, Li-Xin Xiang, Jian-Zhong Shao, Jiong Chen

**Affiliations:** 1 Laboratory of Biochemistry and Molecular Biology, School of Marine Sciences, Ningbo University, Ningbo 315211, China; nieli@nbu.edu.cn; 2 College of Life Sciences, Zhejiang University, Hangzhou 310058, China; shawnlucy@zju.edu.cn (X.-X.X.); xianglx.zju@gmail.com (L.-X.X.); 3 Laboratory for Marine Biology and Biotechnology, Qingdao National Laboratory for Marine Science and Technology, Qingdao 266237, China

**Keywords:** zebrafish NOD2, zebrafish RIG-I, NF-κB signaling, IFN signaling, negative mutual regulation

## Abstract

Nucleotide-binding oligomerization domain-containing protein 2 (NOD2) and retinoic acid-inducible gene I (RIG-I) are two important cytosolic pattern recognition receptors (PRRs) in the recognition of pathogen-associated molecular patterns (PAMPs), initiating innate antibacterial and antiviral signaling pathways. However, the relationship between these PRRs, especially in teleost fish models, is rarely reported. In this article, we describe the mutual regulation of zebrafish NOD2 (*Dr*NOD2) and RIG-I (*Dr*RIG-I) in innate immune responses. Luciferase assays were conducted to determine the activation of NF-κB and interferon signaling. Morpholino-mediated knockdown and mRNA-mediated rescue were performed to further confirm the regulatory roles between *Dr*NOD2 and *Dr*RIG-I. Results showed that *Dr*NOD2 and *Dr*RIG-I shared conserved structural hallmarks with their mammalian counterparts, and activated *Dr*RIG-I signaling can induce *Dr*NOD2 production. Surprisingly, *Dr*NOD2-initiated signaling can also induce *Dr*RIG-I expression, indicating that a mutual regulatory mechanism may exist between them. Studies conducted using HEK293T cells and zebrafish embryos showed that *Dr*RIG-I could negatively regulate *Dr*NOD2-activated NF-κB signaling, and *Dr*NOD2 could inhibit *Dr*RIG-I-induced IFN signaling. Moreover, knocking down *Dr*RIG-I expression by morpholino could enhance *Dr*NOD2-initiated NF-κB activation, and vice versa, which could be rescued by their corresponding mRNAs. Results revealed a mutual feedback regulatory mechanism underlying NOD2 and RIG-I signaling pathways in teleosts. This mechanism reflects the coordination between cytosolic antibacterial and antiviral PRRs in the complex network of innate immunity.

## 1. Introduction

Host defense against pathogen infection is initiated by the recognition of pathogen-associated molecular patterns (PAMPs) through pattern recognition receptors (PRRs). Well-recognized PRRs include toll-like receptors (TLRs), retinoic acid-inducible gene I (RIG-I)-like receptors (RLRs), nucleotide oligomerization domain (NOD)-like receptors (NLRs), C-type lectin receptors (CLRs), and the family of DNA sensors [1,2,3,4,5,6]. Among these PRRs, NOD2 and RIG-I are the two important cytosolic PRRs that focus on the recognition of bacterial and viral PAMPs, respectively. These PRRs initiate signaling pathways that produce proinflammatory cytokines and type I interferons (IFN-Is) to eliminate invading pathogens [7,8].

NOD2 is a member of the NLR family and a general cytoplasmic sensor for most bacteria [9,10,11]. Structurally, NOD2 consists of two N-terminal caspase recruitment domains (CARDs), a central nucleotide-binding domain (NBD), and multiple C-terminal leucine-rich repeats (LRRs). NOD2 is naturally held in an autoinhibited monomeric state by its LRR motif. After recognizing the muramyl dipeptide (MDP), a peptidoglycan (PGN) motif present in all Gram-positive and Gram-negative bacteria, NOD2 oligomerizes through its NBD domain and recruits the downstream adaptor receptor-interacting serine/threonine kinase 2 (RIPK2) via CARD–CARD interactions to activate the NF-κB and MAPK signaling pathways. This activity leads to the production of a series of anti-inflammatory cytokines [12,13]. These processes are important for bacterial clearance. Thus, disrupting these processes increases host susceptibility to various types of bacteria. 

RIG-I is an essential cytoplasmic receptor for recognizing various RNA viruses [14]. This PRR contains two N-terminal CARDs, a central DExD/H box, an RNA helicase domain, and a C-terminal domain (CTD). The CTD of RIG-I has an internal repressor domain (RD), which keeps the RIG-I in an autoinhibited conformation under its resting state [15]. The CARDs act as functional domains for RIG-I signaling, and their overexpression can induce constitutive signaling independent of viral infection [16]. When stimulated by viral RNAs or their corresponding ligands, RIG-I undergoes conformational changes and exposes the CARDs, which then interact with the downstream adaptor mitochondrial antiviral signaling protein (MAVS). These processes initiate antiviral signaling pathways, including the activation of IRF-3/-7 and NF-κB, to produce IFN-Is and proinflammatory cytokines [17,18].

Although NOD2 and RIG-I are important in innate antibacterial/viral processes in the hosts, studies on the correlation between the NOD2 and RIG-I signaling pathways are limited. Previous studies showed that NOD2 could be significantly induced by IFN-β produced through the activation of RIG-I signaling pathway upon viral stimulation [19,20]. However, whether RIG-I could be induced by the NOD2-initiated signaling pathway has not been demonstrated. Moreover, although NOD2 is mostly involved in antibacterial signaling, and RIG-I contributes to antiviral processes, these molecules sometimes share overlapping functionalities [21,22]. These observations imply the coordination between NOD2 and RIG-I signaling pathways during bacterial and viral infections. Clarifying this concept in teleosts can improve the understanding on the regulatory network in innate immunity against different pathogens from an evolutionary perspective.

Teleosts were used in several studies to investigate the structures, signaling pathways, and antipathogen functions of NOD2 and RIG-I [23,24,25,26,27,28,29,30,31,32,33,34,35]. Extensive related studies have been conducted using zebrafish, and the results indicated that *Dr*NOD2 and *Dr*RIG-I were highly conserved and similar to their mammalian counterparts [36,37,38]. Thus, the zebrafish is an attractive model organism to investigate the functional relevance and mutual regulation between RIG-I and NOD2 signaling pathways not only for teleosts, but also for higher species. Expectedly, the results showed that *Dr*RIG-I and *Dr*NOD2 could induce each other, and a close negative mutual regulation between these molecules was observed. Our results provide insights into the coordination of cytosolic antibacterial and antiviral PRRs. This information can improve the understanding on the functional modulation among innate signaling pathways in the complex innate immunity network from an evolutionary perspective.

## 2. Results

### 2.1. Conserved Molecular Pattern of DrRIG-I and DrNOD2

**Figure 1:**
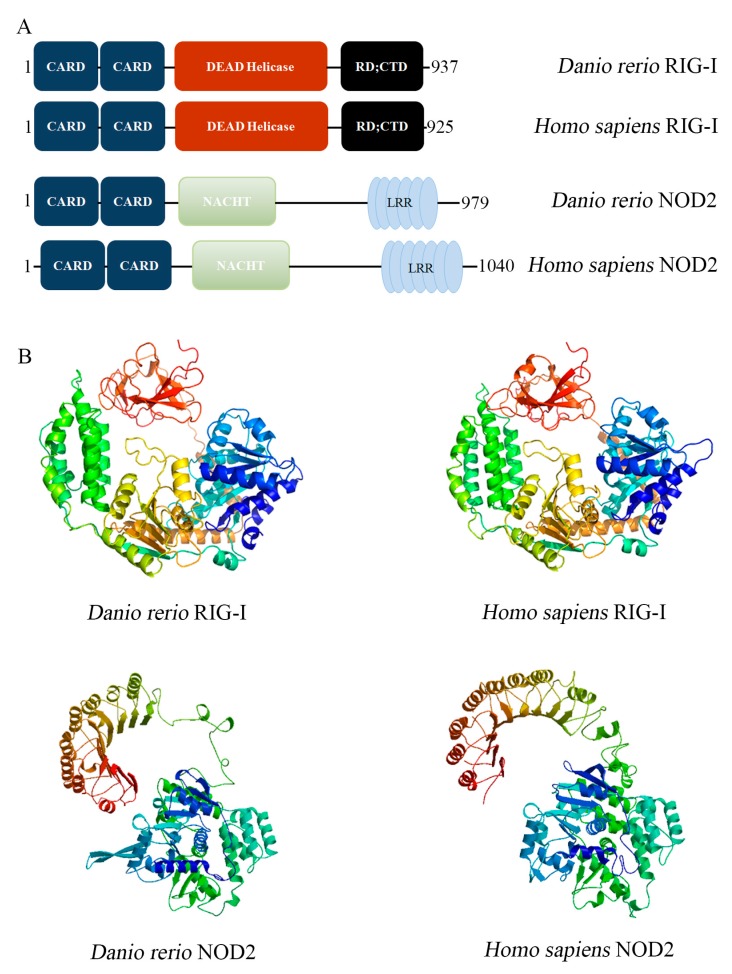
Structural characterization of *Dr*RIG-I and *Dr*NOD2. (**A**) Functional domains and motifs of *Dr*RIG-I, *Homo sapiens* RIG-I, *Dr*NOD2, and *Homo sapiens* NOD2; and (**B**) a comparison of tertiary structures of *Dr*RIG-I and *Dr*NOD2 with their *Homo sapiens* homologs.

We first analyzed the structural conservation of the functional domains of *Dr*RIG-I and *Dr*NOD2 with their mammalian counterparts to investigate the mutual regulation of NOD2 and RIG-I signaling pathways using zebrafish. We used SMART and Pfam databases to refer the protein domains and found that *Dr*RIG-I and *Dr*NOD2 possessed all the functional domains shared by their *Homo sapiens* counterparts (Figure 1A). In homology modeling programs, *Dr*RIG-I and *Dr*NOD2 also showed three-dimensional structures that were highly similar to the RIG-I and NOD2 of *Homo sapiens* (Figure 1B). These findings suggest that RIG-I and NOD2 family members were structurally well-conserved throughout the vertebrate evolution from teleosts to mammals. Hence, our functional study using zebrafish could have universal significance.

### 2.2. Activation of DrRIG-I Signaling Inducing DrNOD2 Expression and Vice Versa

**Figure 2:**
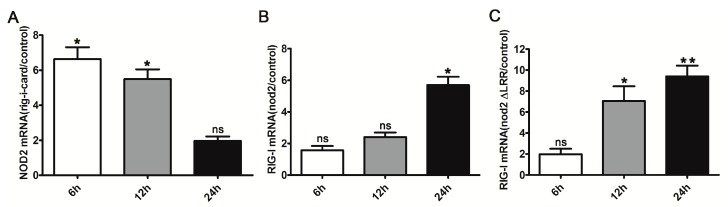
Mutual induction between *Dr*RIG-I and *Dr*NOD2. (**A**) RIG-I signaling activation induced the expression of *Dr*NOD2. One-cell stage embryos were injected with *Dr*RIG-I (CARD) (100 pg/embryo). *Dr*NOD2 was induced 6 and 12 h post injection (hpi), with the highest level at 6 hpi; (**B**,**C**) NOD2 signaling activation induced the expression of *Dr*RIG-I. One-cell stage embryos were injected with *Dr*NOD2 (**B**) and *Dr*NOD2 (ΔLRR) (**C**) (100 pg/embryo). At 24 hpi, the expression of *Dr*RIG-I was upregulated in the *Dr*NOD2-injected group (**B**); At 12 and 24 hpi, RIG-I was induced in the *Dr*NOD2 (ΔLRR) injection group, with higher expression at 24 hpi (**C**). All experiments were conducted with three replicates, and 80–100 zebrafish embryos were collected for the analysis. Values are expressed as mean ± SD; * *p* < 0.05, ** *p* < 0.01.

After confirming *Dr*RIG-I and *Dr*NOD2 conservation structurally, we further investigated the mutual regulation between *Dr*RIG-I and *Dr*NOD2. In mammals, the activation of RIG-I signaling pathway could induce the expression of NOD2. However, whether this induction was conserved in zebrafish and whether *Dr*NOD2 signaling activation induced the expression of *Dr*RIG-I remain unknown. In this experiment, a stimulatory mutant of *Dr*RIG-I containing only CARDs (*Dr*RIG-I-CARD) was used, instead of wild-type *Dr*RIG-I, because the latter remains in an auto-inhibited state without ligand stimulation [37]. In addition to *Dr*NOD2, a mutant without the LRR domain (*Dr*NOD2 (ΔLRR)) was also used to activate the NOD2 signaling. Our results showed that *Dr*NOD2 was induced at 6 and 12 h post injection (hpi) of *Dr*RIG-I (CARD) and reached its highest level at 6 h. Surprisingly, we also observed the induction of *Dr*RIG-I when *Dr*NOD2 signaling was activated at 24 hpi (Figure 2A,B). Moreover, the induction of *Dr*RIG-I could be observed as early as 12 hpi in the *Dr*NOD2 (ΔLRR)-injected group (Figure 2C). Thus, we inferred a feedback regulatory mechanism between these two cytosolic signaling pathways.

### 2.3. DrRIG-I Negative Regulation of DrNOD2-Initiated Signaling

**Figure 3:**
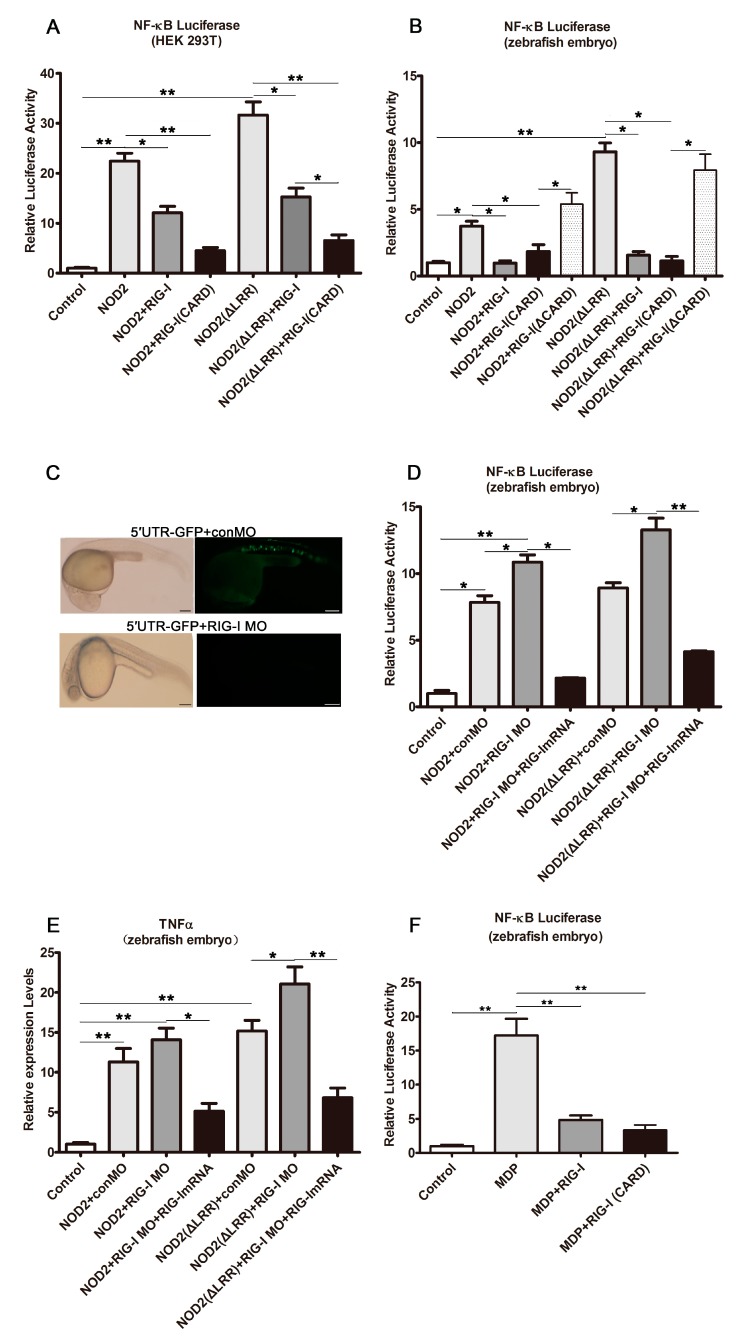
*Dr*RIG-I negative regulation of the *Dr*NOD2-initiated signaling. (**A**,**B**) *Dr*RIG-I negatively regulated *Dr*NOD2-activated NF-κB signaling in HEK293T cells (**A**) and zebrafish embryos (**B**). HEK293T cells (1 µg/mL) or one-cell stage embryos (100 pg/embryo) were administered with *Dr*NOD2 or *Dr*NOD2 (ΔLRR). The cells/embryos were collected at 24 h post transfection/injection. Luciferase assays showed robust NF-κB activation. This activation was evidently inhibited when *Dr*RIG-I or *Dr*RIG-I-CARD was co-administered with *Dr*NOD2 or *Dr*NOD2 (ΔLRR). However, no inhibition was observed in the *Dr*RIG-I (ΔCARD) co-administered group; (**C**) Examination of knockdown efficiency of *Dr*RIG-I MO. The 5′ UTR sequence (complement to the MO sequence) of *Dr*RIG was amplified and inserted into the EGFP-N1 vector. One-cell stage embryos were injected with the constructed vector (100 pg/embryo) and the control MO or *Dr*RIG MO (4 ng/embryo). The embryos were collected at 24 hpi, and phase contrast images and GFP fluorescence were observed to examine the knockdown efficiency, scale bar, 400 µm; (**D**,**E**) The role of *Dr*RIG-I in *Dr*NOD2-initiated signaling was confirmed via MO-mediated knockdown and mRNA rescue in zebrafish embryos. One-cell stage embryos were injected with *Dr*NOD2 or *Dr*NOD2 (ΔLRR) (100 pg/embryo) and control MO or *Dr*RIG MO (4 ng/embryo) or together with *Dr*RIG mRNA (100 pg/embryo). At 24 hpi, NF-κB activation and TNFα production were elevated when *Dr*RIG-I MO was co-administered. Good rescue was achieved with the simultaneous injection of *Dr*RIG-I Mrna; (**F**) *Dr*RIG-I negatively regulated MDP-initiated NOD2 signaling in zebrafish embryos. One-cell stage embryos were administered with 2 nL (1 µg/μL) of MDP. The embryos were collected at 24 hpi. Luciferase assay results showed robust NF-κB activation, which was inhibited by the co-administration of MDP with *Dr*RIG-I or *Dr*RIG-I-CARD. All luciferase assays and qRT-PCR were conducted with three replicates, and each replicate contained the extracts from the cells of a well from the six-well plate or 80–100 zebrafish embryos. Values are expressed as mean ± SD; * *p* < 0.05, ** *p* < 0.01.

To prove our hypothesis, we determined the function of *Dr*RIG-I in *Dr*NOD2-initiated NF-κB activation in the HEK293T cells and zebrafish embryos. The results showed that administration of *Dr*NOD2 or *Dr*NOD2 (ΔLRR) alone led to a robust NF-κB activation in the HEK293T cells (Figure 3A) and zebrafish embryos (Figure 3B). Activation was suppressed when *Dr*RIG-I or *Dr*RIG-I-CARD was co-injected (Figure 3A,B). The mutant without the CARD domain *Dr*RIG-I (ΔCARD) was also used as negative control, and this mutant could not inhibit the *Dr*NOD2- and *Dr*NOD2 (ΔLRR)-activated NF-κB signaling (Figure 3B). The *Dr*RIG-I translation in the zebrafish embryos was knocked down using *Dr*RIG-I MO to confirm the negative role of *Dr*RIG-I in NOD2 signaling. The efficiency of MO was examined before use, and significant knockdown efficiency was observed (Figure 3C). Co-administration *Dr*RIG-I MO resulted in the elevation of the *Dr*NOD2 and *Dr*NOD2 (ΔLRR)-mediated NF-κB activation and TNF-α expression. Good rescue was achieved with the simultaneous injection of *Dr*RIG-I mRNA (Figure 3D,E). MDP-initiated NOD2 signaling was also suppressed when *Dr*RIG-I or *Dr*RIG-I-CARD was co-injected in the zebrafish embryos (Figure 3F). These results demonstrated the negative roles of *Dr*RIG-I in NOD2 signaling. No apparent developmental defects were observed (data not shown).

### 2.4. DrNOD2 Negative Regulation of DrRIG-I-Initiated Signaling

**Figure 4:**
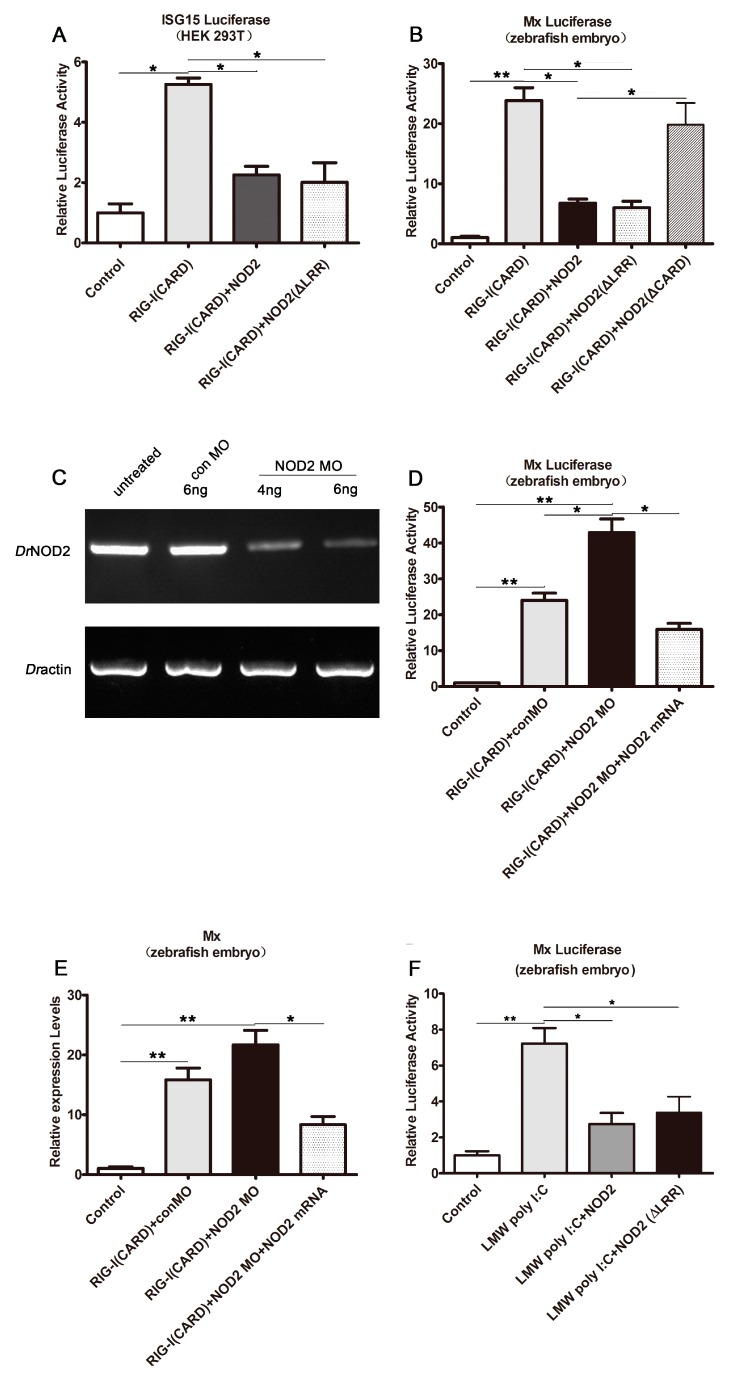
*Dr*NOD2 negative regulation of *Dr*RIG-I-initiated signaling. (**A**,**B**) *Dr*NOD2 negative regulation of *Dr*RIG-I-induced IFN signaling in HEK293T cells (**A**) and zebrafish embryos (**B**); HEK293T cells (1 µg/mL) or one-cell stage embryos (100 pg/embryo) were administered with *Dr*RIG-I (CARD). Cells/embryos were collected at 24 h after transfection/injection. Luciferase assays were conducted to examine the production of ISG15 (HEK293T) and Mx (zebrafish). The results indicated the activation of IFN signaling. The activation of ISG15 or Mx was robust in the *Dr*RIG-I (CARD)-injected group and was suppressed when *Dr*NOD2 or *Dr*NOD2 (ΔLRR) was co-injected, but not the *Dr*NOD2 (ΔCARD) co-injected group; (**C**) Examination of the knockdown efficiency of *Dr*NOD2 MO. One-cell stage embryos of zebrafish were injected with *Dr*NOD2 MO (4 or 6 ng/embryo) or standard control MO (6 ng/embryo). Embryos were collected for RNA isolation and cDNA reverse transcription at 24 hpi. PCR was conducted to detect the deletion of exon 2. The *Dr*NOD2 MO injection groups showed low amplification of exon 2; (**D,E**) Further confirmation of the role of *Dr*NOD2 in *Dr*RIG-I-initiated signaling via MO-mediated knockdown and mRNA rescue in zebrafish embryos. One-cell stage embryos were injected with *Dr*RIG-I (CARD) (100 pg/embryo) and control MO or *Dr*NOD2 MO (4 ng/embryo) or together with *Dr*NOD2 mRNA (100 pg/embryo). Mx activation and expression were elevated when *Dr*NOD2 MO was co-administered. Good rescue was achieved with the simultaneous injection of *Dr*NOD2 mRNA; (**F**) *Dr*NOD2 negative regulation of low-molecular weight (LMW) poly I:C-initiated RIG-I signaling in zebrafish embryos. One-cell stage embryos were administered with 4 nL (1 µg/μL) LMW poly I:C. The embryos were collected at 24 hpi. Luciferase assay results showed robust Mx activation. However, this activation was inhibited when *Dr*NOD2 or *Dr*NOD2 (ΔLRR) was co-administered with LMW poly I:C. All luciferase assays and qRT-PCR were conducted with three replicates, and each replicate contained extracts from the cells of a well from the six-well plate or 80–100 zebrafish embryos. Values are expressed as mean ± SD; * *p* < 0.05, ** *p* < 0.01.

The roles of *Dr*NOD2 in *Dr*RIG-I-induced IFN signaling were also examined. *Dr*RIG-I (CARD) overexpression induced the ISG15 expression in the HEK293T cells (Figure 4A) and Mx in the zebrafish embryos (Figure 4B). ISG15 and Mx are interferon-stimulated genes (ISGs) that indicate the activation of IFN signaling. This activation was suppressed when *Dr*NOD2 or *Dr*NOD2 (ΔLRR) was co-injected (Figure 4A,B). The CARD domain deletion mutant *Dr*NOD2 (ΔCARD) could not inhibit the *Dr*RIG-I (CARD)-initiated Mx activation (Figure 4B). Highly efficient knockdown of *Dr*NOD2 by MO (Figure 4C) resulted in the robust activation and expression of Mx, and simultaneous injection of *Dr*NOD2 mRNA decreased the level of activation (Figure 4D,E). Furthermore, low-molecular weight (LMW) poly I:C-initiated RIG-I signaling was suppressed when *Dr*NOD2 or *Dr*NOD2 (ΔLRR) was co-injected in zebrafish embryos (Figure 4F). These results clearly demonstrated the negative mutual regulation between *Dr*NOD2 and *Dr*RIG-I signaling pathways. No apparent developmental defects were observed (data not shown).

## 3. Discussion

RIG-I and NOD2 are two of the most important cytosolic PRRs participating in the recognition of viral and bacterial invasion in mammals. In the present study, we used zebrafish as an attractive model organism to investigate the NOD2- and RIG-I-mediated immunology. The zebrafish was selected because of its conserved structural and functional characteristics compared with its mammalian counterparts.

We used the zebrafish model to demonstrate that RIG-I signaling activation could induce *Dr*NOD2 expression and that NOD2 signaling activation could induce *Dr*RIG-I expression. These observations suggest a feedback regulatory mechanism between the two signaling pathways. Further evaluation showed that the overexpression of *Dr*RIG-I or *Dr*RIG-I-CARD in the HEK293T cells and zebrafish embryos could significantly inhibit *Dr*NOD2 and *Dr*NOD2 (ΔLRR)-activated NF-κB signaling. Similarly, administration of *Dr*NOD2 or *Dr*NOD2 (ΔLRR) in cells and embryos could significantly suppress *Dr*RIG-I (CARD)-induced IFN signaling. Further knockdown and rescue experiments also confirmed this proposition. Therefore, negative feedback and mutual regulation exist between *Dr*NOD2 and *Dr*RIG-I signaling pathways. Moreover, the results showed the physiological significance of this mutual feedback regulation. *Dr*RIG-I could negatively regulate MDP-initiated NOD2 signaling, and *Dr*NOD2 could negatively regulate LMW poly I:C initiated RIG-I signaling. NOD2 was recently confirmed to interact with RIG-I in a zebrafish cell line (ZF4) by co-immunoprecipitation analysis [38], and we established that this interaction relies on the CARD domain in HEK293T cells (Figure S1). Moreover, the interaction of mammalian NOD2 with RIG-I and mutual regulation have been observed in human cell lines [39]. These descriptions supported our present observations. We believed that the mutual regulation between *Dr*NOD2 and *Dr*RIG-I signaling pathways may have resulted from the sequestering of the CARDs of *Dr*RIG-I and *Dr*NOD2 away from their adaptors, MAVS and RIPK2, respectively.

Therefore, *Dr*NOD2 and *Dr*RIG-I could be induced by their corresponding signaling pathway and negative regulate the signaling. We believe that this feedback might be of great biological significance in innate antibacterial or antiviral immunity. For example, when the host NOD2/RIG-I signaling is activated via bacterial/viral infection, a feedback regulation might contribute to maintain the homeostasis of immune responses. This characteristic prevents excessive immune reactions. Meanwhile, the induced NOD2 or RIG-I would alert the host, leading to a much faster and stronger immune response against a secondary bacterial or viral invasion. Thus, dysfunction in this feedback regulation may result in various pathological disorders. Secondary bacterial infection commonly develops after viral infections, and this process is accompanied by bursts of inflammatory responses [19]. In addition, susceptibility to bacterial super infection is usually increased as a consequence of innate antiviral responses [40]. We infer that these observations might be associated, at least partially, with the cross-regulation between NOD2 and RIG-I signaling pathways. However, the exact underlying mechanisms of these processes require further clarification. A comprehensive understanding of the cross-regulation between antibacterial and antiviral signaling pathways has long been a challenging task because of the limited availability of research models. Our findings on the mutual regulation between NOD2 and RIG-I signaling pathways in zebrafish may provide a basis for elucidating the cross-regulatory mechanisms of different innate immune signaling pathways. Finally, our work may also be beneficial for the study on the coordination among innate immune systems from the evolutionary perspective.

## 4. Materials and Methods

### 4.1. Experimental Fish

Wild-type AB zebrafish (*Danio rerio*) weighing 0.5–1 g and measuring 1–2 cm in length were purchased from the National Zebrafish Resources of China. The fish were kept in tanks with recirculating water at 28 °C and fed daily with commercial pellets at 0.7% of their body weight. The fish were acclimatized and evaluated for overall fish health at least two weeks before the experiments.

### 4.2. Bioinformation Analyses

The *Dr*NOD2 sequence was retrieved from the National Center for Biotechnology Information [41], and the *Dr*RIG-I sequence was obtained from a previous study [37]. Functional domains and motifs in proteins were analyzed using SMART and Pfam databases [42,43]. Tertiary structures were determined through PyMOL [44].

### 4.3. Plasmid Constructions

The open-reading frame (ORF) of *Dr*NOD2 was inserted into pCMV-HA (Beyotime, Shanghai, China) between the EcoRI and XhoI sites to construct the eukaryotic expression vector (pCMV-*Dr*NOD2). The LRR motif-deleted mutant (ΔLRR) construct (pCMV-*Dr*NOD2 (ΔLRR)) and the CARD motif-deleted mutant (ΔCARD) construct (pCMV-*Dr*NOD2 (ΔCARD)) were cloned and inserted into the pCMV-HA between the sites of EcoRI and XhoI. The CARD motif-deleted mutant (ΔCARD) construct of *Dr*RIG-I was cloned and inserted into the pcDNA6 between the sites of BamHI and EcoRI. The NF-κB luciferase vector was purchased from Clontech (Palo Alto, CA, USA), while the pRL-TK vector was obtained from Promega (Madison, WI, USA). Human ISG15 and zebrafish Mx promoters (hISG15-pro-luc and *Dr*Mx-pro-luc), as well as the RIG-I expression plasmids pcDNA6-*Dr*RIG-I and pcDNA6-*Dr*RIG-I (CARD), were constructed in our previous study [37,45]. All primers used in plasmid construction are shown in Table S1.

### 4.4. Morpholino Oligonucleotide (MO) and Capped mRNA

The translation-blocking MOs of *Dr*RIG-I and splice junction MO of *Dr*NOD2 were designed, synthesized by Gene Tools (Philomath, OR, USA), and dissolved in water (2 mM). The MO sequences used were as follows: *Dr*RIG-I MO, 5′-GATTCTCCTTCTCCAGCTCGTACAT-3′; and *Dr*NOD2 MO, 5′-ACCTGCCAAAAATCCAACATGGTTA-3′. The 5′UTR sequence (complement to the MO sequence) of *Dr*RIG was amplified with *Dr*RIG-I F1 and R1 primers (Table S1), and then cloned into the EGFP-N1 vector to evaluate the translation blocking efficiency of *Dr*RIG-I MO. Along with *Dr*RIG-I MO or standard control MO (4 ng/embryo), the constructed vector was injected into one-cell stage embryos (100 pg/embryo). The embryos were collected at 24 hpi, and the GFP fluorescence was visualized through an Olympus MVX10 MacroView. The splice inhibition efficiency of *Dr*NOD2 MO was examined through RT-PCR. The one-cell stage embryos of zebrafish were injected with *Dr*NOD2 MO (4 or 6 ng/embryo) or standard control MO (6 ng/embryo). The embryos were collected at 24 hpi for RNA isolation and cDNA reverse transcription. A forward primer in exon 1 and reverse primer in exon 2 were used to detect the deletion of exon 2 (Table S1). Capped *Dr*RIG-I and *Dr*NOD2 mRNA was synthesized in vitro using a Message Machine kit (Ambion, Thermo Fisher Scientific, Waltham, MA, USA) according to the manual and then solubilized in DEPC water.

### 4.5. Cell Culture and Transient Transfection

HEK293T cells were maintained in Dulbecco’s modified Eagle’s medium (DMEM, Biochrom AG, Berlin, Germany) supplemented with 10% (*v*/*v*) FBS (Gibco, Thermo Fisher Scientific, Waltham, MA, USA), penicillin (100 U/mL), and streptomycin (100 µg/mL) at 37 °C in 5% CO_2_. Cells were seeded into six-well plates to allow growth until 70–90% confluence on the day of transfection. Transient transfection and cell lysate preparation were performed as previously described [45].

### 4.6. Luciferase Assay

HEK293T cells or zebrafish embryos were transfected (1 µg/mL) or injected (100 pg/embryo) with relative stimulant plasmids and NF-κB/ISG15/Mx luciferase reporter vectors. The pRL-TK renilla luciferase reporter plasmid was used as internal control. An empty control plasmid was then added to ensure same amounts of the total DNA. Subsequently, the cells and embryos were lysed at 24 hpi, and dual-luciferase reporter assay was performed as described previously [46]. Luciferase activity was normalized to pRL-TK activity and expressed as fold stimulation relative to the control.

### 4.7. Induction Assay

pcDNA6-*Dr*RIG-I (CARD) or pCMV-*Dr*NOD2 or pCMV-*Dr*NOD2 (ΔLRR) (100 pg/embryo) was injected into one-cell stage embryos. The mRNA levels of NOD2 or RIG-I relative to β-actin were examined at 6, 12, and 24 hpi using RT-PCR to determine whether RIG-I-initiated signaling induced NOD2 production and vice versa. The empty plasmid injection group was set as control. RT-PCR was conducted with the following parameters: (1) 40 cycles of amplification at 95 °C for 30 s and 60 °C for 20 s; (2) melting curve analysis at 95 °C for 5 s, 65 °C for 15 s, and 95 °C for 15 s; and (3) cooling at 40 °C for 30 s. Relative gene expression was calculated using the 2^−∆∆*C*t^ method with NOD2/RIG-I, which were initially normalized against β-actin. The primers used are shown in Table S1. Each PCR trial was performed in triplicate and repeated at least thrice.

### 4.8. Co-Immunoprecipitation Assay

pcDNA6-*Dr*RIG-I (CARD) (Myc tag) and pCMV-*Dr*NOD2 (HA tag) were transfected into HEK293T cells. At 48h post transfection, cells were lysed with cold lysis buffer (1% Triton X-100, 150 mM NaCl, 1 mM EDTA, 20 mM Tris-HCl (pH 7.4)) containing protease inhibitor mixture (Roche, Basel, Switzerland) for 30 min at 4 °C. lysates were centrifuged for 15 min at 14,000 rpm and the supernatants were incubated with mouse anti-HA Ab (Abcam, Cambridge, MA, USA) at 4 °C overnight and then incubated with protein A-agarose beads (Roche) for 4 h. The obtained samples were subjected to Western blot assays using rabbit anti-c-Myc tag and HRP-conjugated goat anti-rabbit IgG Ab (Abcam) antibodies, and visualized with ECL reagents as described before.

### 4.9. Mutual Regulation between DrNOD2 and DrRIG-I

The mutual regulation between *Dr*NOD2 and *Dr*RIG-I was examined in the HEK293T cells and zebrafish embryos. The role of *Dr*RIG-I in the *D*rNOD2-activated NF-κB signaling was analyzed by administering pCMV-*Dr*NOD2 or pCMV-*Dr*NOD2 (ΔLRR), alone or together, with pcDNA6-*Dr*RIG-I, pcDNA6-*Dr*RIG-I (CARD), or pcDNA6-*Dr*RIG-I (ΔCARD) and the NF-κB luciferase reporter plasmid and internal control plasmid pRL-TK to the HEK293T cells or one-cell stage zebrafish embryos. The CARD deletion mutant was used as the negative control. The role of *Dr*NOD2 in *Dr*RIG-I-induced IFN signaling was analyzed by administering pcDNA6-*Dr*RIG-I (CARD), alone or together, with pCMV-*Dr*NOD2, pCMV-*Dr*NOD2 (ΔLRR), or pCMV-*Dr*NOD2 (ΔCARD). The IFN luciferase reporter plasmid (hISG15-pro-luc in HEK293T and *Dr*Mx-pro-luc in embryo) and control plasmid pRL-TK were also administered. The CARD deletion mutant was used as a negative control. The cells/embryos were harvested and lysed at 24 h post transfection/injection for dual-luciferase reporter assay.

### 4.10. Morpholino-Mediated Knockdown and Capped mRNA-Mediated Rescue

Knockdown and rescue experiments were conducted to further confirm the roles of *Dr*RIG-I in *Dr*NOD2-initiated pathways and *Dr*NOD2 in *Dr*RIG-I*-*initiated pathways. For the former, one-cell stage embryos were injected with pCMV-*Dr*NOD2/pCMV-*Dr*NOD2 (ΔLRR) (100 pg/embryo) and *Dr*RIG-I MO (4 ng/embryo), or together with capped *Dr*RIG-I mRNA (100 pg/embryo). For the latter, one-cell stage embryos were injected with pcDNA6-*Dr*RIG-I (CARD) (100 pg/embryo) and *Dr*NOD2 MO (4 ng/embryo) or together with capped *Dr*NOD2 (100 pg/embryo) mRNA. The embryos in each group were harvested at 24 hpi and lysed for dual-luciferase reporter assay. Furthermore, qRT-PCR was conducted to examine the relative expression levels of TNFα (for NF-κB signaling) and Mx (for IFN signaling).

### 4.11. Role of DrRIG-I and DrNOD2 in MDP-Initiated NOD2 Signaling and LMW Poly I:C-Initiated RIG-I Signaling

We further confirmed the physiological significance of the mutual negative regulatory roles between *Dr*RIG-I and *Dr*NOD2 by evaluating the role of *Dr*RIG-I in MDP-initiated NOD2 signaling and *Dr*NOD2 in LMW poly I:C-initiated RIG-I signaling. For *Dr*RIG-I, one-cell stage embryos were administered with 2 nL (1 µg/μL) of MDP, alone or together, with pcDNA6-*Dr*RIG-I or pcDNA6-*Dr*RIG-I (CARD) (100 pg/embryo), as well as NF-κB luciferase reporter plasmids. The embryos were collected at 24 hpi, and luciferase assays were conducted to examine the NF-κB activation level. For *Dr*NOD2, one-cell stage embryos were administered with 4 nL (1 µg/μL) of LMW poly I:C alone or together with pCMV-*Dr*NOD2/pCMV-*Dr*NOD2 (ΔLRR) (100 pg/embryo), as well as Mx luciferase reporter plasmids. The embryos were collected at 24 hpi, and luciferase assays were conducted to examine Mx activation level.

### 4.12. Statistical Analysis

Data from three independent experiments were expressed as mean ± SD. Groups were compared statistically using Student’s *t*-test for paired samples. Statistical significance were considered at * p < 0.05 and ** p < 0.01.

## Supplementary Materials

The following are available online at http://www.mdpi.com/1422-0067/18/6/1147/s1.

## Abbreviations

NOD2	nucleotide oligomerization domain 2
RIG-I	retinoic acid-inducible gene I
PRRs	pattern recognition receptors
PAMPs	pathogen-associated molecular patterns
MDP	muramyl dipeptide
TLRs	toll-like receptors
RLRs	RIG-I-like receptors
NLRs	NOD-like receptors
CLRs	C-type lectin receptors
IFN-Is	type I interferons
CARDs	caspase activation and recruitment domains
NBD	nucleotide-binding domain
LRRs	leucine-rich repeats
PGN	peptidoglycan
RIPK2	receptor-interacting serine/threonine kinase 2
CTD	C-terminal domain
RD	repressor domain
MAVS	mitochondrial antiviral signaling protein
DrNOD2	*Danio rerio* NOD2
DrRIG-I	*Danio rerio* RIG-I
ORF	open reading frame
DMEM	Dulbecco’s modified Eagle’s medium
LMW poly I:C	low molecular weight poly I:C
