# Supplementary material for: Mutual Regulation of NOD2 and RIG-I in Zebrafish Provides Insights into the Coordination between Innate Antibacterial and Antiviral Signaling Pathways
Source: Int J Mol Sci. 2017 May 27;18(6):1147. doi: 10.3390/ijms18061147 (PMC5485971; doi:10.3390/ijms18061147)
Supplement: Supplementary file 1 [file ijms-18-01147-s001.pdf]

Table S1. Primers used for plasmid construction and gene expression analysis

Primer name	Sequence (5’→3’)	Use
*Dr*NOD2 F1	ATGAACGCTCAACAGTTGATCCTCAAAC	Gene cloning
*Dr*NOD2 R1	GAAGGTCAATCTGCATTCTTGCTGGCTC	Gene cloning
*Dr*NOD2 F2	CCGGAATTCATGAACGCTCAACAGTTGA	Plasmid construction
*Dr*NOD2 R2	CCGCTCGAGGAAGGTCAATCTGC	Plasmid construction
*Dr*NOD2 ΔLRR R	CCGCTCGAGACACGGCAGAAGCTGCTCCACA	Mutant construction
*Dr*NOD2 ΔCARD F	CCGCTCGAGAAAGAAAATACACCTTCCGC	Mutant construction
*Dr*RIG-I ΔCARD F	CGCGGATCCGACTGCATGATGGCTGAATCC	Mutant construction
*Dr*RIG-I ΔCARD R	CCGGAATTCCAGTTGACCAGCGCCCATGTCT	Mutant construction
*Dr*RIG-I F1	GCCACCATGTACGAGCTGGAGAAGGAGAATCTG ATGGTGAGCAAGGGCGAGGA	Plasmid construction
*Dr*RIG-I R1	TTACTTGTACAGCTCGTCCATGCCGA	Plasmid construction
*Dr*NOD2 F3	GGTAATGGATGCGTTATGGTGGGCAAGGACA	Exon2 verification
*Dr*NOD2 R3	GATTGGTGAGGTTGGCTCAGGTTGC	Exon2 verification
*Dr*RIG-I qrt F	CGGACCTCAGTTTCAAGG	Real-time PCR
*Dr*RIG-I qrt R	GCAGCGGGAGAATATGGA	Real-time PCR
*Dr*NOD2 qrt F	CGAGCAGGGCTTCTGGCAGTTCTAT	Real-time PCR
*Dr*NOD2 qrt R	GCTCAATCGCAGCCAAAAAATAACT	Real-time PCR
*Dr*β-actin qrtF	ACACCTTCTACAATGAGCTG	Real-time PCR
*Dr*β-actin qrtR	CTGCTTGCTGATCCACATCT	Real-time PCR

F, forward primer; R, reverse primer.

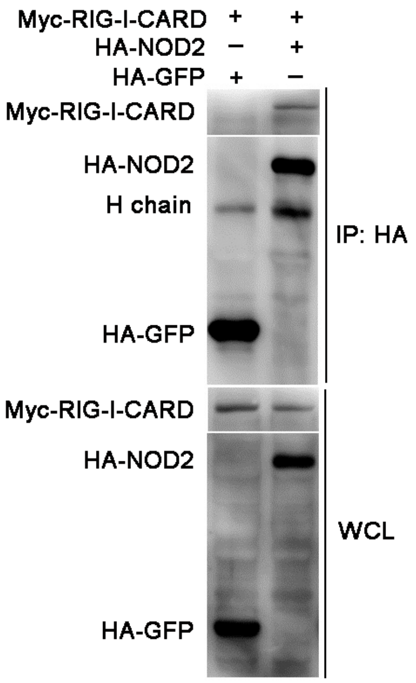

Figure S1. Interaction of DrNOD2 and DrRIG-I (CARD) was evaluated by Co-IP. pcDNA6-*Dr*RIG-I (CARD) (Myc tag) and pCMV-*Dr*NOD2 (HA tag) were transfected into HEK293T cells. At 48h post transfection, cells were lysed and the supernatants were incubated with mouse anti-HA Ab at 4℃ overnight and then incubated with protein A-agarose beads for 4h. The obtained samples were subjected to western-blot assays using rabbit anti-c-Myc tag and HRP-conjugated goat anti-rabbit IgG Ab antibodies, and visualized with ECL reagents.
